# Naturally russeted and wound russeted skins of mango (cv. ‘Apple’) show no differences in anatomy, chemical composition or gene expression

**DOI:** 10.1038/s41598-025-86563-1

**Published:** 2025-01-18

**Authors:** Jannis Straube, Thomas O. Athoo, Viktoria Zeisler-Diehl, Kiran Suresh, Lukas Schreiber, Moritz Knoche

**Affiliations:** 1 Institute of Horticultural Production Systems, Fruit Science Section, Leibniz University Hannover, Herrenhäuser Straße 2, 30419 Hannover, Germany; 2 Institute of Cellular and Molecular Botany (IZMB), Department of Ecophysiology, University of Bonn, Kirschallee 1, 53115 Bonn, Germany

**Keywords:** Periderm, Cuticle, Russeting, *Mangifera indica*, Wounding, Developmental biology, Plant sciences

## Abstract

The mango cultivar ‘Apple’ is commercially important in Kenya but highly susceptible to russeting. Russeting refers to an area of fruit skin where the primary (epidermal) surface has been replaced by a secondary (peridermal) surface. The objective was to establish histologies, gene expressions and chemical compositions of a natural periderm, a wound-induced periderm and of cuticles of an un-russeted skin. Fluorescence microscopy revealed a suberized phellem in natural and wound-induced periderms. Wound-induced periderms had more cell layers and a higher mass than natural periderms. Compared with cuticles, periderms showed decreased expressions of cuticle-related genes (*MiSHN1*,* MiGPAT6*, *MiCUS1*,* MiCER1*,* MiWCB11*) and increased expressions of periderm-related genes (*MiMYB93*, *MiNAC058*,* MiCYP86A1*, *MiCYP86B1*, *MiGPAT5*,* MiABCG20)*. Natural periderms and wound-induced periderms contained cutin and suberin monomers (C16-C28), including carboxylic, dicarboxylic, and ω-hydroxy acids and primary alcohols. Cuticles of the primary skin contained cutin monomers of similar chemistry but shorter chain lengths (C16-C22). The wax composition in natural and wound-induced periderms was similar to that in cuticles. Lignin monomers in natural and wound-induced periderms contained *p*-hydroxyphenyl, guaiacyl and syringyl units, but only traces of these occurred in cuticles. In ‘Apple’ mango, the histologies, gene expressions and compositions of natural and wound-induced periderms are largely the same.

**Supplementary Information:**

The online version contains supplementary material available at 10.1038/s41598-025-86563-1.

## Introduction

The mango cultivar ‘Apple’ is commercially important in Kenya, but it is also very susceptible to russeting, an unattractive surface disorder that occurs in many fruit crops around the world^1–5^. Anatomically, russeting involves the formation of a secondary skin (periderm), just beneath a compromised primary skin (epidermis). Functionally, a periderm (multicellular) partially reestablishes the barrier properties lost following damage to an epidermis (one-cell layer thick). A periderm comprises a thin meristematic phellogen layer, which divides to produce a thin layer of parenchymatous tissue - a phelloderm (to the inside) and a thicker layer of loosely-stacked cork cells - a phellem (to the outside). The cell walls of the phellem are impregnated with the waterproofing polymers lignin and suberin. This corky layer is responsible for the unattractive, dull-brown appearance of a russeted fruit surface. Compared with a primary skin, a periderm has increased permeability to water, so russeting accelerates postharvest mass loss and can lead to shrivel^6–8^.

Russeting (the development of a periderm) is commonly triggered when the cuticle is ruptured by microscopic cracks (microcracks). Mechanical damage^9,10^, high humidity^11,12^ and persistent surface moisture are all factors that can increase microcracking and thus induce russeting^8,10,12–17^. Young fruit are particularly susceptible to russeting because their relative rates of surface expansion are especially high. These high relative growth rates, cause significant growth strain^18,19^. Microcracks form, when high rates of surface expansion are not matched by correspondingly high rates of cuticle deposition^13,14,20^. Microcracks impair the barrier functions of the cuticle, and it is this impairment that triggers the formation of a periderm in the hypodermal layer, usually just beneath an area of microcracking^13,21–23^.

In cv. ‘Apple’ mango, microcracking and, hence, russeting tends to begin in the vicinity of lenticels^1,24^. Incidence is especially high following extended periods of surface wetness and/or low night temperatures^1,24^. In contrast, in *Malus* apple, lenticels do not seem to play a role in the russeting but mechanical wounding of the fruit skin does cause russeting^10^. Although russeting in cv. ‘Apple’ mango seems to be triggered by somewhat different factors from those triggering russeting in *Malus* apple^1,24^, we postulate that surface wounding may also be a factor in russeting of ‘Apple’ mango.

We are unaware of any information on the molecular background of russeting in mango. Periderm induction is a highly variable process that is largely affected by environmental factors. This makes systematic studies on russeting difficult. We therefore tested whether mechanical wounding could serve as a proxy for russet induction under field conditions. This approach could minimize the impact of environmental variation on russet formation and provide a useful tool for studying the molecular processes involved in russet development.

The objectives of our study were: (1) to compare the histologies of naturally russeted skins of fruit of cv. ‘Apple’ mango, with those of skins showing wound-induced russeting and with un-russeted primary skins (cuticles); (2) to quantify the expressions of genes associated with cuticle and periderm formation; and (3) to analyse the chemical compositions of naturally induced periderms, wound-induced periderms and cuticles.

## Results

**Fig. 1:**
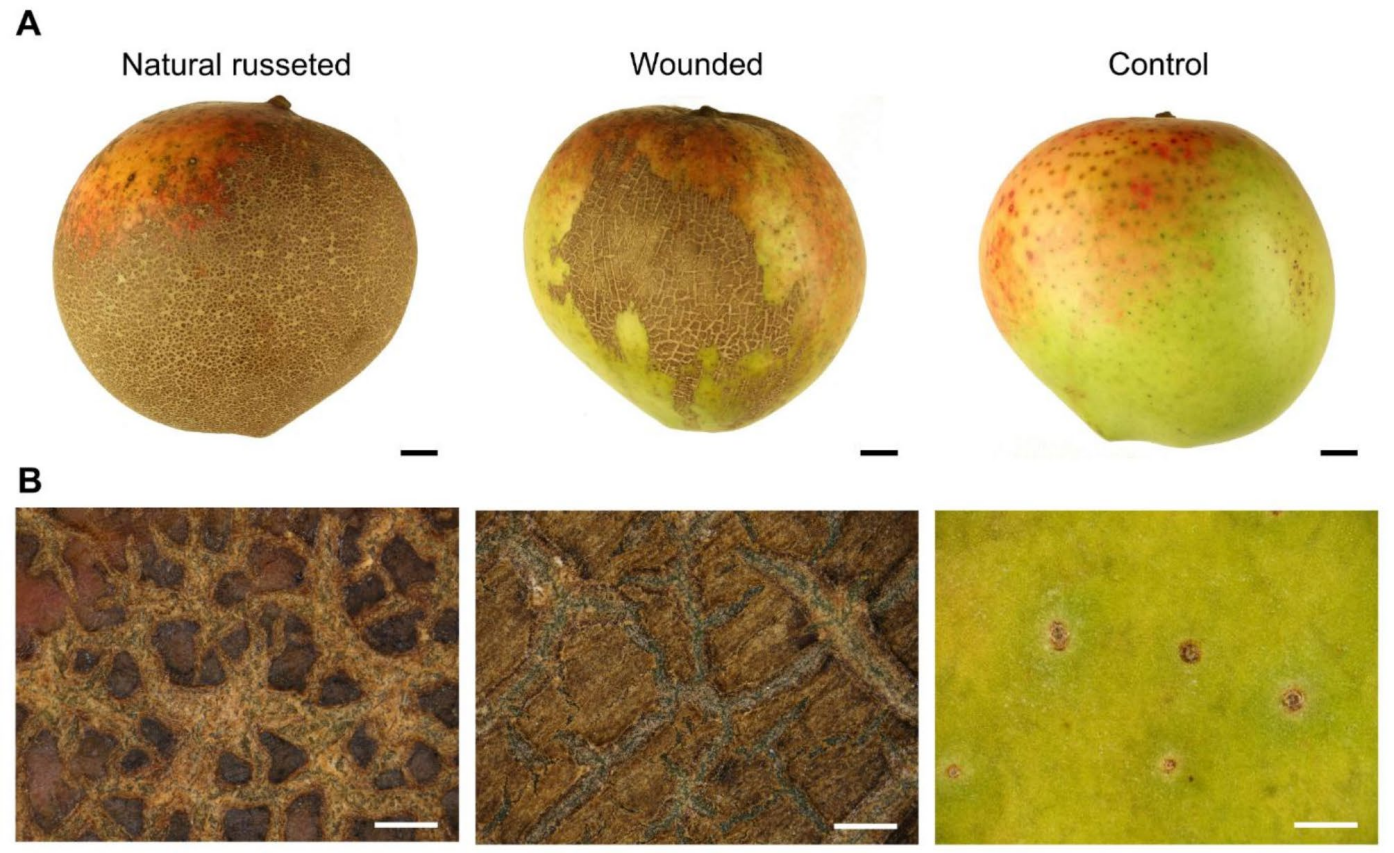
Macroscopic (**A**) and microscopic views (**B**) of periderms on naturally russeted fruit skins and on mechanically wounded fruit skins and of epidermises on un-russeted (control) fruit skins of ‘Apple’ mango at maturity. For mechanical wounding (‘Wounded’), a fruit skin was lightly abraded with sandpaper 60 days after full bloom (DAFB). The opposite side of each wounded fruit was left without wounding as a control (‘Control’). Fruit were sampled at maturity (126 DAFB). Scale bars in (**A**) = 1 cm, scale bars in (**B**) = 1 mm.

Visually, the russeted surfaces of cv. ‘Apple’ mango fruit appeared brownish and dull, compared with the smooth, shiny surfaces of un-russeted fruit (Fig. 1A). Light microscopy revealed that, natural russet and wound-induced russet both showed a reticulated pattern of cuticular microcracking in the russeted areas. No comparative structures were evident in un-russeted fruit surfaces (Fig. 1B).

**Fig. 2:**
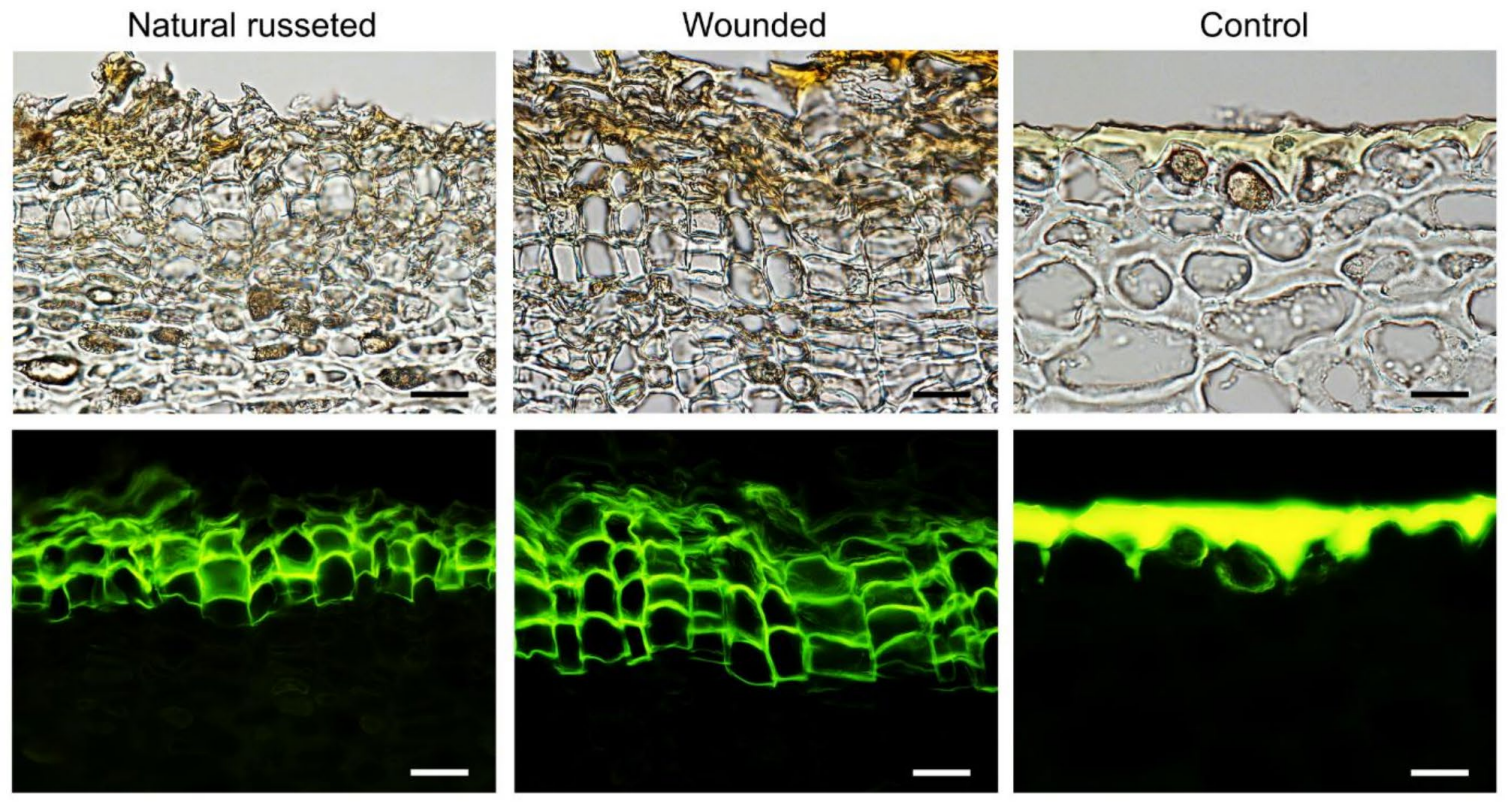
Cross-sections through skin samples with periderms on naturally russeted fruit skins, with periderms resulting from mechanical wounding and with intact epidermises (‘control’) of ‘Apple’ mango at maturity (126 days after full bloom (DAFB)). For mechanical wounding, fruits skins were lightly abraded using sandpaper 60 days after full bloom (DAFB) (‘Wounded’). The opposite side of each wounded fruit was left without wounding as a control (‘Control’). Micrographs were taken under transmitted white light or incident fluorescent light after staining with Fluorol Yellow 088 (scale bars 20 µm).

Fluorescence microscopy using fluorol yellow as a dye revealed the stacks of suberized cells typical of a phellem, in both natural and wound-induced periderms (Fig. 2). A wound-induced periderm appeared to be more organized and to have more cell layers in the phellem, than a natural periderm (Fig. 2; Table 1). Compared with a natural russeted periderm, a wound-induced periderm was more compact (Fig. 2). Cross-sections through an un-russeted fruit surface revealed the cuticle to be variable in thickness, and to have deep pegs above the anticlinal cell walls of the epidermis.

**Table 1:** Mass of cuticular membrane (CM), peridermal membrane (PM), dewaxed cuticular membrane (DCM) or dewaxed peridermal membrane and of wax per unit area on periderms of naturally russeted fruit skins, periderms resulting from mechanical wounding and non-peridermal fruit skins (‘control’) of ‘Apple’ mango at maturity (126 days after full bloom (DAFB)). The wax content (%) was calculated by dividing the difference between the CM and DCM masses per unit area by the CM mass and multiplying by 100 (*n* = 10). The number of phellem cell layers was determined by counting the number of suberized cell walls that stained with Fluorol Yellow 088 (*n* = 6). Data represent means ± SE. Means followed by the same letter are not significantly different according to Tukey HSD, at *p* < 0.05.

Treatment		Mass per unit area (g·m^–2^)				Number of cell layers in phellem
		Controls	Dewaxed	Wax	Wax content (%)	
Natural russeted	PM	40.2^a^ ± 1.7	37.4^a^ ± 1.7	2.7^a^ ± 0.2	6.8	3.0^a^ ± 0.1
Wounded	PM	65.6^b^ ± 3.6	62.8^b^ ± 3.5	2.8^a^ ± 0.3	4.3	3.8^b^ ± 0.1
Control	CM	13.5^c^ ± 0.2	11.2^c^ ± 0.2	2.3^a^ ± 0.0	16.8	0.0^c^ ± 0.0

The mass of a periderm isolated from a wounded fruit skin was higher than that from a naturally russeted fruit skin (Table 1). Compared to both types of periderm, the mass of cuticle per unit area was lowest. The mass of wax per unit area of cuticle was not different between a wounded fruit skin and a naturally russeted one.

**Fig. 3:**
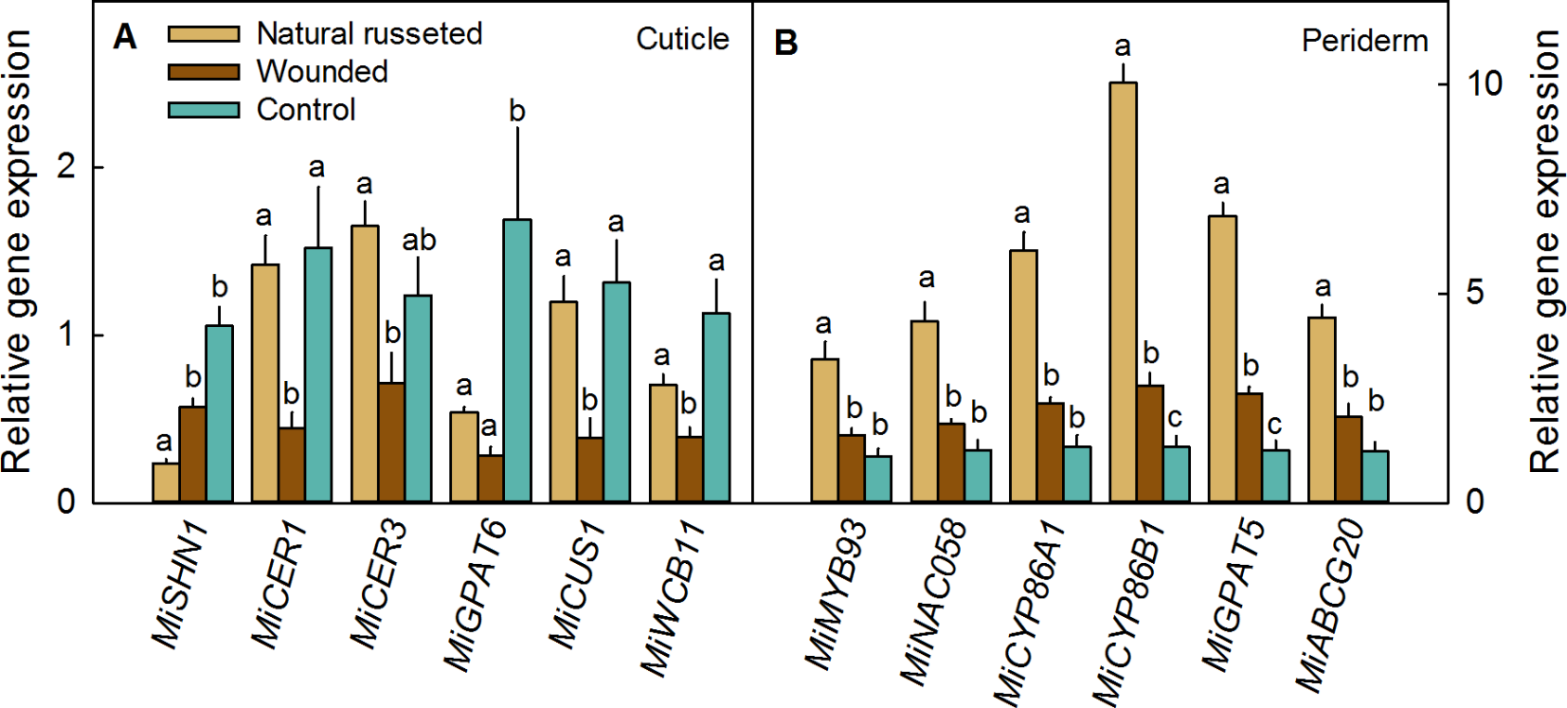
Expression of cuticle-related (**A**) and periderm-related genes (**B**) in skin samples with periderms on naturally russeted fruit skins, with periderms resulting from mechanical wounding and with intact epidermises (‘control’) of ‘Apple’ mango at maturity (126 days after full bloom (DAFB)). For wounding, fruits skins were lightly abraded using sandpaper at 60 days after full bloom (DAFB) (‘Wounded’). The opposite side of each wounded fruit was left without wounding as a control (‘Control’). Expressions of genes related either to the cuticle (*MiSHN1*, *MiWBC11*, *MiCUS1*, *MiCER1*, *MiCER3*, *MiGPAT6*) (**A**) or to the periderm (*MiMYB93*, *MiNAC058*, *MiCYP86A1*, *MiCYP86B1*,* MiGPAT5*, *MiABCG20*) (**B**) were analyzed. Gene expression values represent means ± SE of three independent biological replicates. Columns identified by the same letter do not differ significantly, one way ANOVA post-hoc Tukey HSD at *p* < 0.05.

Russeted patches of skin showed decreased expressions of cuticle-related genes (Fig. 3A). Specifically, those genes involved in regulating cuticle formation (*MiSHN1*), the synthesis of cutin (*MiGPAT6*, *MiCUS1*) and wax (*MiCER1*), and the transport of cutin monomers and wax constituents (*MiWCB11*) were all downregulated in both naturally russeted and wound russeted fruit skins.

The expressions of periderm-related genes, including those involved in transcriptional regulation (*MiMYB93*, *MiNAC058*), suberin synthesis (*MiCYP86A1*, *MiCYP86B1*, *MiGPAT5*), and suberin monomer transport (*MiABCG20*), were all upregulated in naturally russeted fruit skins compared to un-russeted controls. In wound russeted skins, the expressions of some periderm related genes were also upregulated, but this was significant only for *MiCYP86B1* and *MiGPAT5* at maturity (Fig. 3B).

**Fig. 4:**
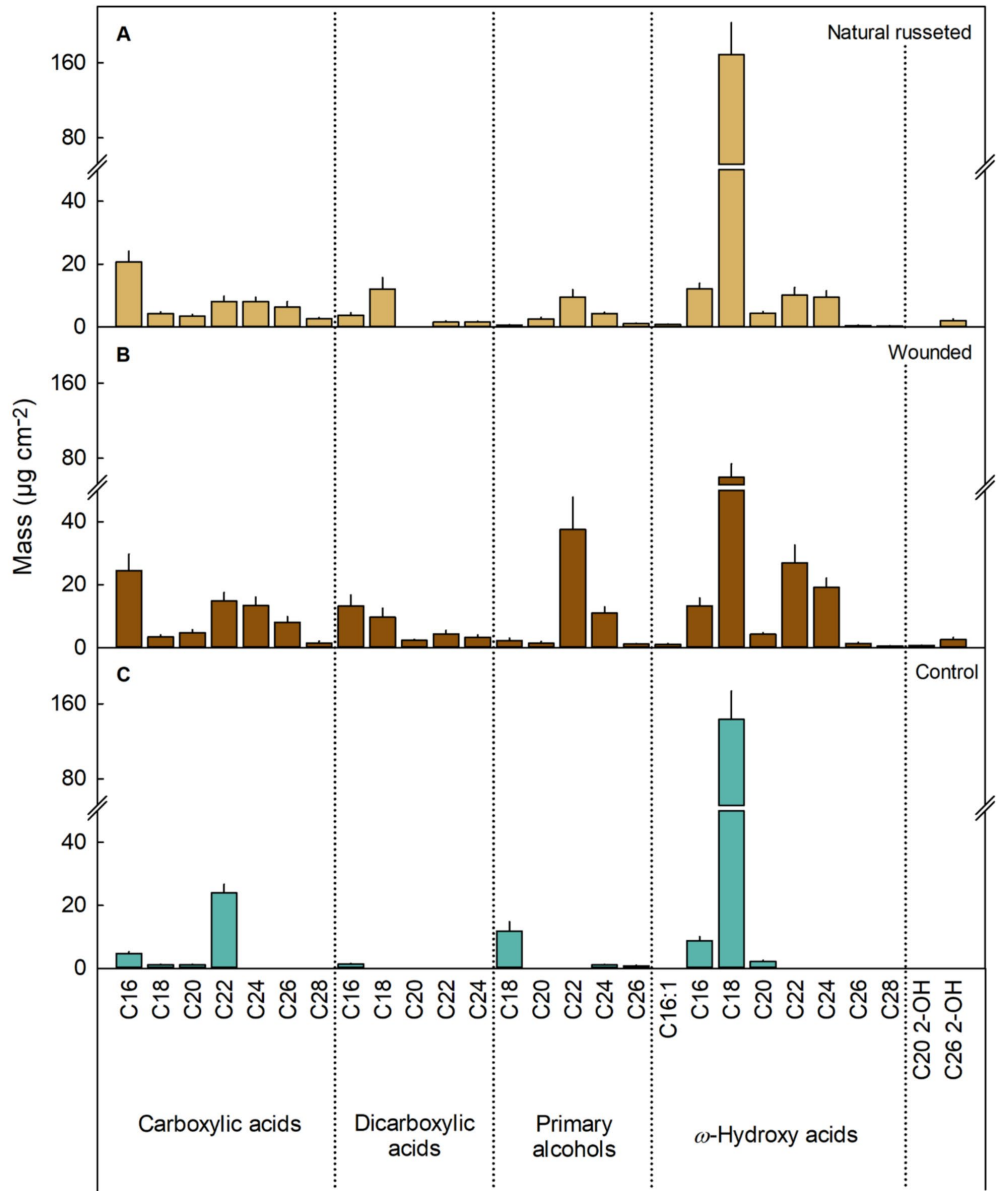
Cutin and suberin monomers in periderms on naturally russeted fruit skins, on periderms resulting from mechanical wounding and intact epidermises (‘control’) of ‘Apple’ mango at maturity (126 days after full bloom (DAFB)). For wounding, fruits skins were lightly abraded using sandpaper at 60 days after full bloom (DAFB) (‘Wounded’). The opposite side of the wounded fruit was left without wounding as a control (‘Control’). Data represent the mean ± SE of five independent biological replicates each consisting of five disks of cuticular membrane (CM) or of peridermal membrane (PM).

The periderm membranes (PMs) of naturally russeted and wound russeted skins contained various cutin and suberin monomers, including carboxylic acids, dicarboxylic acids, primary alcohols, and *ω*-hydroxy acids, with carbon chain lengths ranging from C_16_ to C_28_ (Fig. 4A, B). In contrast, the cuticles of the un-russeted controls were composed of cutin monomers, mainly carboxylic and *ω*-hydroxy acids, typically with chain lengths of C_16_ to C_22_, and with fewer dicarboxylic acids and primary alcohols (Fig. 4C). The 18-hydroxy stearic acid was the most common and abundant monomer in both cuticular membranes (CMs) and PMs (Fig. 4A-C). C_24_ to C_28_ carboxylic acids, C_18_ to C_24_ dicarboxylic acids, C_20_ and C_22_ primary alcohols and C_22_ to C_28_*ω*-hydroxy acids were exclusively found in russeted skins and were totally absent in un-russeted ones (Fig. 4A-C). A comparison of naturally russeted and wound russeted skins revealed higher levels of suberin-specific *ω*-hydroxy acids and primary alcohols with carbon chain lengths of C_22_ and C_24_ in the wound russeted skins. Wound russeted skins had lower amounts of 18-hydroxy stearic acid than either naturally russeted or un-russeted skins (Fig. 4A, B).

**Fig. 5:**
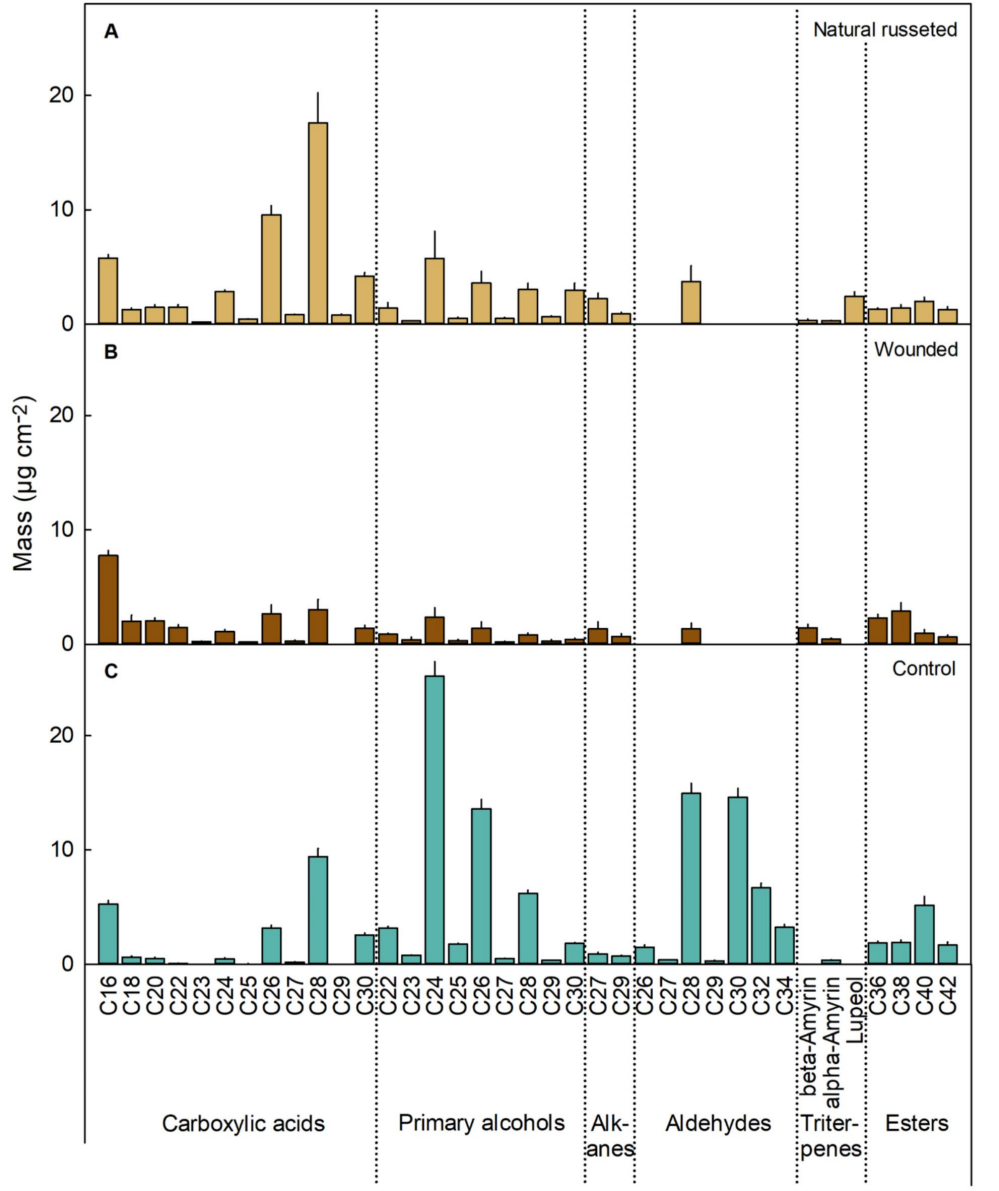
Wax constituents in periderms of naturally russeted fruit skins, periderms resulting from mechanical wounding and in non-peridermal skin patches (‘control’) of cv. ‘Apple’ mango at maturity (126 days after full bloom (DAFB)). For wounding, fruit skins were abraded using sandpaper at 60 days after full bloom (DAFB) (‘Wounded’). The opposite side of the wounded fruit was left without wounding and served as control (‘Control’). Data represent the means ± SE of five independent biological replicates consisting of five CM/PM disks each.

Wax constituents identified in natural russeted PMs and wound-induced russeted PMs comprised C_16_ to C_30_ carboxylic acids, C_22_ to C_30_ primary alcohols, C_27_ and C_29_ alkanes, and esters ranging from C_36_ to C_42_, along with C_28_ aldehydes and triterpenes like lupeol, *α*- and *β*-amyrin (Fig. 5A, B). The un-russeted controls had similar compositions i.e., C_16_ to C_30_ carboxylic acids, C_22_ to C_30_ primary alcohols, C_27_ and C_29_ alkanes, C_26_ to C_34_ aldehydes, and esters ranging from C_36_ to C_42_ plus traces of the triterpene *α*-amyrin (Fig. 5C). The most abundant constituents in russeted skins were C_16_, C_26_, and C_28_ carboxylic acids, but those in un-russeted skins were C_24_ and C_26_ primary alcohols and C_28_ and C_30_ aldehydes. The main compositional difference between russeted and un-russeted skins was the absence of C_26_, C_27_, C_29_, C_30_, C_32_ and C_34_ aldehydes in the PMs (Fig. 5A-C). Lupeol was present only in natural russeted skins (Fig. 5A, B).

**Fig. 6:**
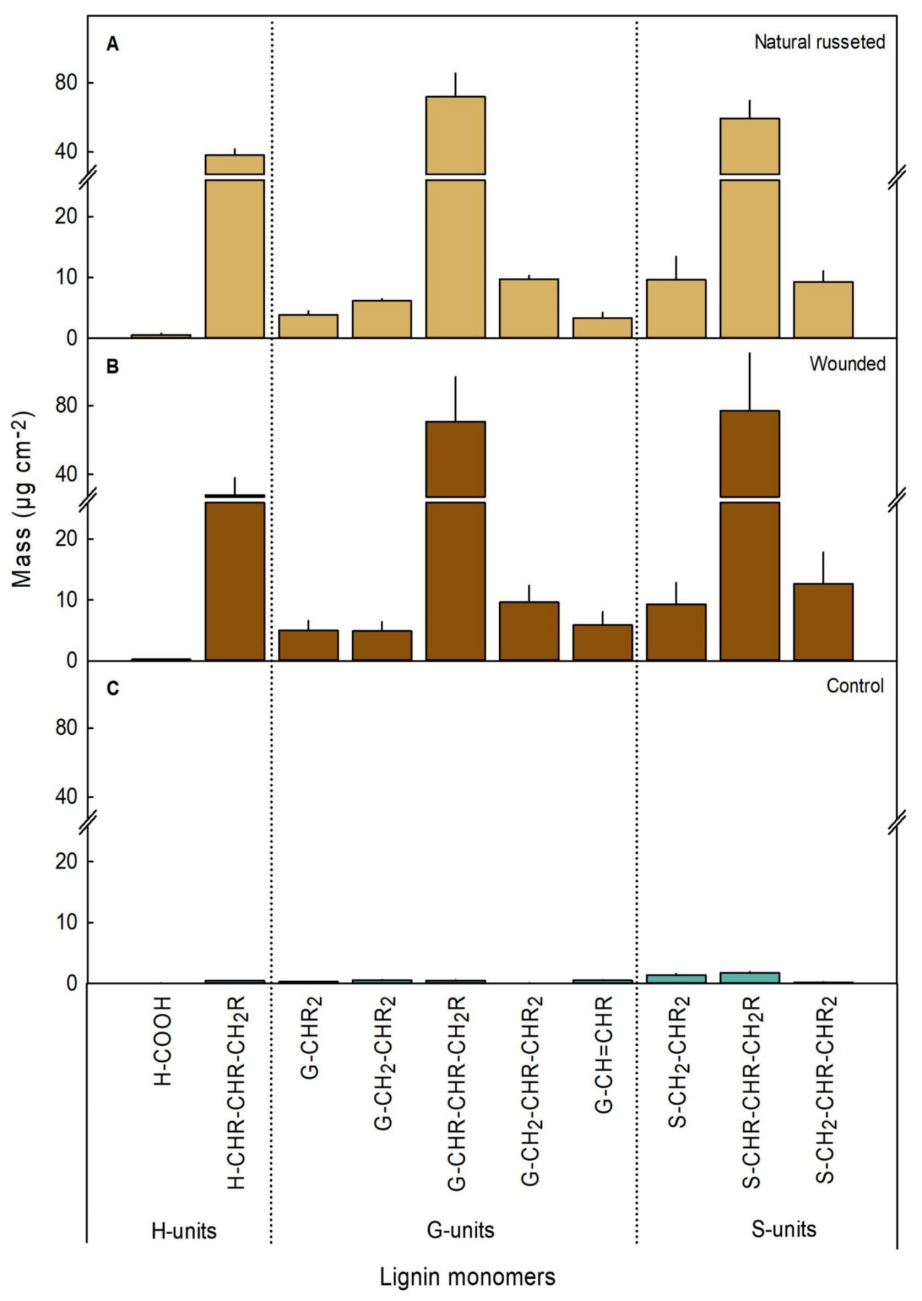
Lignin monomers in periderms on naturally russeted fruit skins, periderms resulting from mechanical wounding and in non-peridermal skin patches (‘control’) of cv. ‘Apple’ mango at maturity (126 days after full bloom (DAFB)). For wounding, fruit skins were abraded using sandpaper at 60 days after full bloom (DAFB) (‘Wounded’). The opposite side of the wounded fruit was left without wounding and served as control (‘Control’). The H-, G-, and S-units are shown as peaks identified in the GC-MS spectra as described by Rolando et al.^59^. (H: p-Hydroxyphenyl (4-hydroxyphenyl); G: Guaiacyl (4-hydroxy-3-methoxyphenyl); S: Syringyl (4- hydroxy-3,5-dimethoxyphenyl); R: EtS (Ethanthiol)). Data represent the means ± SE of four to five independent biological replicates consisting of five CM/PM disks each.

Analysis of lignin monomers by thioacidolysis in the PMs of naturally russeted and wound russeted skins yielded a variety of *p*-hydroxyphenyl (H), guaiacyl (G), and syringyl (S) units products (Fig. 6A, B). The G-units (95.1 ± 13.3 µg/cm^2^) were most common in the lignin of naturally russeted skins (Fig. 6A), while both G- (96.17 ± 12.9 µg/cm^2^) and S-units (98.9 ± 22.0 µg/cm^2^) were detected in similar amounts in wound russeted skins (Fig. 6B). The monomer G-CHR-CHR-CH_2_R (C_6_C_3_ guaiacyl derivative) was the most common monomer in naturally russeted PMs and S-CHR-CHR-CH_2_R (C_6_C_3_ syringyl derivative) in wound PMs (Fig. 6A, B). In contrast, the CMs of un-russeted skin patches had only traces of H-, G-, and S-units. In the CMs, the S-units were the most abundant (3.4 ± 0.5 µg/cm^2^) with S-CHR-CHR-CH_2_R (C_6_C_3_ syringyl derivative) being the most common (Fig. 6C).

## Discussion

### Periderms of wound russeted fruit are similar to those of naturally russeted fruit but have more cell layers of suberized phellem

The histology and chemical composition of periderms with wound-induced russeting, and those with natural russeting, were generally similar. In each case, a periderm was formed in the hypodermis immediately below an area of a cuticle with impaired barrier properties. Whether the barrier properties were impaired due to microcracking of the cuticle or due to mechanical wounding made no difference. Under field conditions moisture induced microcracking at lenticels is the most common source of natural russeting in ‘Apple’ mango^8,24^. Moisture is also a critical factor in russeting of *Malus* apple^13,19^. Mechanical wounding is caused by contact with neighboring fruit or branches. Periderms caused by natural russeting and by wounding revealed the characteristics typical of a periderm, with only slight differences. Wound-induced periderms had a higher number of layers of phellem cells and a more organized structure below the phellem. This structure presumably represents a region of non-suberized phellem, phellogen and phelloderm. A larger number of layers of phellem cells and/or a greater mass of lignin are consistent with the greater mass of periderm per unit area for a wound-induced periderm.

The chemical composition of the two periderms also showed similarities, including high lignin contents and the presence of suberin-specific monomers such as C_20_, C_22_ and C_24_*ω*-hydroxy acids^14,25,26^. In wound-induced periderms, there was a slight increase in the levels of C_22_ and C_24_*ω*-hydroxy acids and primary alcohols, compared to those of natural periderms. This observation is consistent with that of a greater number of phellem cell layers in the wound-induced periderms. Additionally, wound-induced periderms showed a decrease in the amounts of cutin associated monomers, such as the C_18_ primary alcohols and C_18_*ω*-hydroxy acids, while the periderms of naturally russeted fruit exhibited minimal changes in these components.

Comparing the composition of both periderms with un-russeted skins revealed substantial differences. The un-russeted skins lacked most lignin monomers as well as the suberin specific monomers C_22_ and C_24_*ω*-hydroxy acids and primary alcohols. Lower levels of the wax related primary alcohols and aldehydes were also measured in both periderms than in the un-russeted controls. This finding is consistent with earlier reports in *Malus* apples^10^ and in potato tubers^27^. Comparing the wax compositions of un-russeted skins of the russet-susceptible ‘Apple’ mango with an unsusceptible mango cultivar revealed reduced levels of terpenoids in the russet-susceptible one^28^.

### Expression patterns of periderm related genes are similar in wound-induced russeting and natural russeting

The periderm-associated genes were expressed more strongly in wound-induced periderms and in naturally russeted skins, than in the un-russeted control areas.

Expression of the transcriptional regulators *MiMYB93* and *MiNAC058* associated with periderm formation was increased. In *Malus* apple, the ortholog *MdMYB93* regulates suberin synthesis^29^ and *MdNAC058* is upregulated early in russet formation^14^. Also, *NbNAC058* is upregulated when *MdMYB93* is transiently overexpressed in *Nicotiana benthamiana* leaves^29^. At present, the role of *NAC058* in periderm formation is not clear.

Genes involved in suberin synthesis were increased in both wound-induced and natural periderms as indexed by the upregulation of *MiCYP86A1*, *MiCYP86B1* and *MiGPAT5*. *AtCYP86A1* plays a significant role in the formation of aliphatic suberin formation in *Arabidopsis*, particularly in the synthesis of *ω*-hydroxyacids with a carbon chain length < C20 ^30^. Knockout of *AtCYP86B1* in *A*. *thaliana* led to a reduction in C_22_ and C_24_ fatty acids, indicating the involvement of *AtCYP86B1* in the synthesis of *ω*-hydroxy-C_22_ and -C_24_ acids as well as that of *α*,*ω*-dicarboxylic acids^31^. Additionally, knockout of *AtGPAT5* resulted in a decrease in very long-chain dicarboxylic acid and *ω*-hydroxy acids, which are typical monomers found in aliphatic suberin^32^.

The upregulation of *MiABCG20* implies increased transport of suberin monomers, consistent with the function of its ortholog *AtABCG20* in *Arabidopsis*^33^.

*MiCYP86B1* and *MiGPAT5* were expressed at significant levels in our wound-induced periderms, while in periderms of naturally russeted fruit all periderm-related genes were significantly upregulated. Differences in the expression patterns of periderm-related genes may be accounted for by varying proportions of suberized phellem. The phellem is dead when functional and the larger proportion of phellem in wound-induced periderms may simply have diluted out the tissue that actively expressed genes.

Cuticle related genes were downregulated. The cuticle-associated genes *MiCER1*, *MiCER3*, *MiCUS1* and *MiWCB11* were downregulated only in areas of wound-induced russeting, not in naturally russeted areas. *MiSHN1* and *MiGPAT6* were downregulated in both naturally russeted and wound-induced areas compared with in un-russeted control areas. SHINE transcription factors play important roles in regulating cuticle formation^34,35^. For example, *AtSHN1* regulates cutin and wax synthesis in *Arabidopsis*^36^.

*MiGPAT6* and *MiCUS1* play roles in cutin synthesis, similar to that of *AtGPAT6* in *Arabidopsis*, where it synthesizes 2-monoacylglycerols^37^. A mutation in the related gene *SlGPAT6* in tomatoes reduces cutin content thus leading to thinner cuticles^38^. Additionally, *MdGPAT6* was significantly downregulated in moisture-induced russeting, in wound-induced russeting and in naturally russeted skins of *Malus* apple^10,14,15,39^. Lastly, *SlCUS1* is involved in cutin synthesis and deposition in tomatoes^40–42^.

*MiCER1* and *MiCER3* are involved in the synthesis of very long chain alkanes found in waxes, as shown for *AtCER1* and *AtCER3* in *Arabidopsis*^43–45^.

Similarly, *MiWBC11* is involved in the transport of cutin monomers and wax constituents, as is its ortholog *AtABCG11* in *Arabidopsis*^46^. Consistent with these observations is the downregulation of *MdABCG11* during russet formation in *Malus* apple^14^, which has been identified earlier in a quantitative trait loci (QTL) linked to russeting^47^.

Variation in the extent of downregulation of cuticle related genes may be expected between wound-induced and natural russeting. For both types of russeting, the surface comprises a composite made up partly of peridermal cells and partly of epi- and hypodermal cells and cuticle.

## Conclusion

Our results demonstrate that in ‘Apple’ mango, the histology, gene expression and chemical composition of the periderms of russeted fruit surfaces are broadly the same whether they are wound-induced or natural. In each case, consistent with current views on the etiology of russeting^7^, the periderm is formed as a result of an impaired barrier function, resulting either from cuticular microcracking or a wound. From a scientific point of view mechanical wounding is a useful tool to induce russeting experimentally.

## Methods

### Plant material and treatment

Trees of ‘Apple’ mango (*Mangifera indica* L.) grafted on local seedling rootstocks were grown in a commercial orchard in Kaiti, Kenya (1°45’ S 37°28’ E) in line with local integrated crop management programs^48^. A wound periderm was induced in the cheek region of blemish-free fruit at 60 DAFB by carefully abrading the fruit skin using sandpaper (grit size 1000; Bauhaus, Mannheim, Germany). The area of wounded skin remained unprotected until maturity. An un-abraded area on the opposite side of the same fruit served as control. Naturally russeted fruit were selected randomly. All fruit were harvested at commercial maturity 126 DAFB.

### Macroscopic and microscopic observations

Photographs of whole fruit were taken with a camera (Canon EOS 550D, lens: EF-S 18–55 mm, Canon Germany, Krefeld, Germany). Micrographs of selected areas of skin were taken using a dissecting microscope (Leica MZ10F, Leica Microsystems GmbH, Wetzlar, Germany) fitted with a camera (DP73; Olympus; Tokio, Japan).

Skin segments were excised, fixed in Karnovsky solution and held at 4 °C pending processing for microscopy^49^. For microscopy, skin segments were rinsed in deionized water, transferred to 70% (v/v) aqueous ethanol for 16 h and then dehydrated in an ascending series of ethanol (70%, 80%, 90%, 96% (v/v); each once and for 30 min), followed by isopropanol (100% (v/v), twice, each for 30 min). The dehydrated samples were transferred to xylene substitute (AppliClear; AppliChem, Münster, Germany; twice, each for 30 min), and subsequently to a 1/1 (v/v) mixture of xylene substitute/paraffin for 40 min at 65 °C, followed by melted paraffin at 65 °C (twice, each for 40 min). All dehydration and infiltration steps were supported by reduced pressure (10.8 kPa). Skin segments were then transferred to metal molds, cast in melted paraffin and stored at 4 °C.

Anticlinal sections of the fruit skins (10 μm thickness) were cut using a rotatory microtome (Hydrax M 55; Carl Zeiss, Oberkochen, Germany). Sections were then transferred to microscope slides and dried at 40 °C for 16 h. The paraffin was then removed by xylene substitute (twice, each for 10 min) and rehydrated using a descending ethanol series (96%, 80%, 70% (v/v); once, each for 10 min), followed by deionized water (twice, each for 10 min). The sections were stained using Fluorol Yellow 088 (0.005% (w/v); Santa Cruz Biotechnology, TX, USA) dissolved in a mixture (1/1 (v/v)) of glycerol (90%, (v/v); Carl Roth) and polyethylene glycol 4000 (SERVA Electrophoresis, Heidelberg, Germany) for 1 h^50^. Sections were rinsed in deionized water and viewed under transmitted white light or incident fluorescent light (filter U-MWB; 450–480 nm excitation; >520 nm emission wavelength; Olympus) using a fluorescence microscope (BX-60, Olympus, Hamburg, Germany). In total, six biological replicates were observed for each treatment.

Macrographs and micrographs were stitched using multiple images taken along the z-axis using Helicon Focus (v 7.7.5) software (Helicon Soft Ltd., Kharkiv, Ukraine).

### RNA isolation from fruit skins

Skin samples that exhibited a periderm arising from natural russeting or from wound-induced russeting, or un-russeted controls were excised using a razor blade. Samples were immediately frozen in liquid nitrogen and stored at -80 °C until processing. Samples with wound-induced russeting or un-russeted control samples were taken from opposing sides of the same fruit. Samples showing natural russeting were excised from different fruit of the same batch. RNA was isolated using the InviTrap Spin Plant RNA Mini Kit (STRATEC Molecular GmbH, Berlin, Germany) and RP buffer according to the manufacturer’s protocol. Subsequently, genomic DNA was removed from the RNA using DNase (DNA-free™ Kit; Thermo Fisher Scientific, Waltham, Massachusetts, USA). The quantity and quality of RNA was checked using a Nanodrop 2000c spectrophotometer (Thermo Fisher Scientific, Waltham, MA, USA) at 230, 260, and 280 nm. The integrity of the RNA was investigated on a 1.5% agarose gel. The cDNA was synthesized using the LunaScript^®^ RT SuperMix Kit (New England Biolabs, Ipswich, MA, USA) and 600 ng of total RNA in a reaction volume of 40 µL according to the manufacturer’s protocol.

### Gene expression analysis

Putative genes involved in cuticle formation were identified through a transcriptomic study^51^. Candidate genes associated with periderm related functions were determined via a tblastn search from characterized orthologous genes of *Arabidopsis thaliana* (*AtABCG20*, *AtGPAT5*, *AtCYP86A1*, *AtCYP86B1*) or *Malus x domestica* (*MdMYB93*,* MdNAC058*), as well as one cuticle associated gene of *Arabidopsis thaliana* (*AtGPAT6*). The best hits were taken for further analysis. The tblastn search was done using the Mango RNA-Seq database (http://bioinfo.bti.cornell.edu/cgi-bin/mango/index.cgi)^51^ with the following parameter settings: E-value 1e-10; description: 20; alignment: 20. Additional primers for cuticle associated genes were taken from Tafolla-Arellano et al.^51^. Gene specific primers were designed using the Primer3 software (Primer3, http://primer3.ut.ee/). The reference genes used in this study were *MiActin1*^52^ and *MiTUBB*^53^. All primers are listed in Table S1.

Quantitative real-time PCR was used to determine gene expression (QuantStudio™ 6 Flex Real-Time PCR System; Applied Biosystems, Waltham, MA, USA). The PCR reactions were done using 1 µL of cDNA, 3 µL primer solution (final conc. 200 nM) and 4 µL of Luna^®^ Universal qPCR Master Mix (New England Biolabs, Ipswich, MA, USA) according to the manufacturer’s guidelines. The following PCR conditions were used: 95 °C for 60 s; 40 cycles of 95 °C for 15 s and 60 °C for 60 s. Subsequently melting curves were analyzed to determine specificity of amplifications using the following conditions: 95 °C for 15 s, 60 °C for 60 s and 60–95 °C, in 0.5 °C increments. Primer efficiency was determined using a 1:4 dilution series of a cDNA pool with four dilution points. Efficiency values were calculated using the QuantStudio™ Real-Time PCR Software v1.3 (Applied Biosystems, Waltham, MA, USA). Calculation of differential gene expression was done according to Vandesompele et al.^54^ and Hellemans et al.^55^.

### Isolation of fruit cuticle and periderm membranes

Cuticular membranes (CMs) and periderm membranes (PMs) were isolated enzymatically^56^ from areas of natural russeting, wound-induced russeting and un-russeted controls. Discs were punched using a biopsy punch (12 mm diameter; Acuderm, Terrace, FL, USA). The excised skin discs were incubated in 50 mM citric acid buffer at pH 4.0, pectinase (9%, v/v; Panzym Super E flüssig; Novozymes A/S, Krogshoejvej, Bagsvaerd, Denmark), cellulase (cellulase (0.5% v/v; Cellubrix L.; Novozymes A/S), and 30 mM NaN_3_ at room temperature. The enzyme solution was replaced periodically until the CMs or PMs separated from their subtending tissues. Thereafter, CMs and PMs were rinsed in deionized water, dried at 40 °C for 20 h, and stored at room temperature.

The CMs and PMs were dewaxed using a Soxhlet apparatus and a mixture of chloroform/methanol (1/1 (v/v)). Wax mass was determined by subtracting the mass of the dewaxed CM from that of the CM.

### Wax constituent identification and quantification

Five CM or PM discs, each excised from a different fruit, were cut into small pieces and pooled to create a single CM or PM replicate. Wax was extracted overnight from 1.0 to 2.0 mg of CM or 4.0 to 8.5 mg of PM in 5 ml CHCl_3_ at room temperature on a horizontal rolling bench (CAT RM. 5–30 V, Staufen, Germany). Tetracosane was added as an internal standard (100 µL of 10 mg tetracosane in 50 mL CHCl_3_) for quantification. The volume of CHCl_3_ was gradually reduced under a gentle stream of N_2_ at 60 °C. The dewaxed CMs and PMs were then dried on Teflon discs and held at room temperature for analysis of their cutin and suberin monomer compositions.

The wax extracts were derivatized by silylation, yielding the corresponding trimethylsilyl ethers and esters. Briefly, 20 µL BSTFA (N, O-bis(trimethylsilyl)-trifluoroacetamid; Macherey-Nagel, Düren, Germany) and 20 µL pyridine (Sigma Aldrich; Deisenhofen, Germany) were added to each sample followed by incubation for 45 min at 70 °C. Wax constituents were quantified by on-column injection using a gas chromatograph (GC) equipped with a flame ionization detector (FID) (GC-Hewlett Packard 5890 series H, Hewlett-Packard, Palo Alto, CA, USA) and a 30 m DB-1 column (0.32 mm i.d., film 0.1 μm; J&W Scientific, Folsom, CA, USA). The wax constituents were identified by GC-MS (Quadrupole mass selective detector HP 5971, Hewlett-Packard, Palo Alto, CA, USA). For quantification the internal standard was used. For identification of constituents the fragmentation patterns were compared with literature data and an in-house data library. Four to five replicates were run, each replicate consisted of five CM/PM disks, each from a different fruit.

### Identification and quantification of cutin and suberin monomers

The dewaxed CMs and PMs were transesterified by incubation in 1 mL of BF_3_/MeOH for 16 h at 70 °C. Subsequently, an internal standard of 20 µg of dotriacontane (100 µL of 10 mg dotriacontane in 50 mL CHCl_3_) was added. The depolymerization of cutin and suberin was stopped by adding 2 mL of saturated NaHCO_3_. Cutin and suberin monomers were extracted using 3 × 2 mL CHCl_3_. The CHCl_3_ phase containing suberin/cutin monomers was washed with 1 mL of HPLC-grade water, dried with Na_2_SO_4_ and reduced under a gentle stream of N_2_ at 60 °C. Subsequently, samples were derivatized according to the aforementioned procedure. Quantitative analysis of monomers and constituents was carried out using a GC-FID equipped with a splitter injection system, the identification was done using a GC-MS as described above. Data were normalized relative to the internal standard and expressed per unit area of fruit surface. Five replicates were run, with each replicate consisting of five CM or PM discs, each from a different fruit.

### Lignin monomer identification and quantification

For chemical analysis of lignin, the mango periderms were cut into small pieces and depolymerized by thioacidolysis. For this, protocols from Foster et al.^57^ and Qi et al.^58^ were used with slight modifications. One PM per sample was incubated in 500 µL of the thioacidolysis reagent, in an autosampler vial, on a heating block (105 °C) for 4 h. The vials were sealed with an extra Teflon disc inside the crimp seal with PTFE/silicone septa to prevent evaporation of the solvent and burning of the samples. Samples were vortexed once per hour. Upon completion of the reaction, autosampler vials containing the samples were cooled to room temperature and spiked with 10 µg of C_32_ (Dotriacontane) as an internal standard. To stop the reaction, 500 µL of saturated NaHCO_3_ solution was added. Lignin monomers were extracted three times with 1 mL of ethyl acetate. The combined organic extracts were dried in a heating block at 60 °C, using a gentle nitrogen stream and 1 mL of acetone was added twice to remove all excess water. Samples were finally derivatized with 20 µL pyridine and 100 µL BSA reagent (N, O-bis(trimethylsilyl)acetamide, Sigma Aldrich, Missouri, USA) for 45 min at 70 °C. Lignin monomers were quantified by injecting 1 µL of sample on a splitter system of a GC equipped with an FID (HP 6890 N, Hewlett-Packard, Palo Alto, California, USA). Lignin monomers were identified by GC-MS (GC-MSD; 5977B, Agilent, Santa Clara, USA) according to Rolando et al.^59,60^ thioacidolysis products prominent fragments.

### Data analysis and presentation

Data are presented as means $\:\pm\:$ standard errors. Statistical comparisons were conducted using RStudio (R version 3.6.1; R Foundation for Statistical Computing, Vienna, Austria;^61^). The data were analyzed by one-way ANOVA with a post-hoc Tukey HSD test (*p* < 0.05).

## Electronic supplementary material

Below is the link to the electronic supplementary material.

Supplementary Material 1

Supplementary Material 2

## Data Availability

All data supporting the findings of this study are available within the manuscript or supplementary information files.
