# Supplementary material for: Naturally russeted and wound russeted skins of mango (cv. ‘Apple’) show no differences in anatomy, chemical composition or gene expression
Source: Sci Rep. 2025 Jan 18;15:2366. doi: 10.1038/s41598-025-86563-1 (PMC11742678; doi:10.1038/s41598-025-86563-1)
Supplement: Supplementary file 1 — Supplementary Material 1 [file 41598_2025_86563_MOESM1_ESM.docx]

**Table S1.** Cuticle and periderm specific primers used in this study

|  | | | | | | | | | |
| --- | --- | --- | --- | --- | --- | --- | --- | --- | --- |
|  |  | | **Primer sequence (5'-3')** | | |  | |  | |
| **Gene name** | | **Accession** | | **Forward Primer** | **Reverse Primer** | | **PCR efficiency (%)** | **Reference** | |
|  | |  | |  |  | |  |  | |
| **Cuticle related** | |  | |  |  | |  |  | |
| *MiSHN1* | | MIN047952 | | GGCTCTTGGGTCTCTGAG | CCTCTTCAGCCGTCTCAA | | 90.3 | Tafolla-Arellano et al. 2017 ^51^ | |
| *MiWBC11* | | MIN106958 | | GAGATAGAGACGAGCAAG | CTCCCACAAGTTCTGTATTAG | | 93.6 | Tafolla-Arellano et al. 2017 ^51^ | |
| *MiCER1* | | MIN107433 | | GATTGTTTCTACCACTTAACACC | CACCCTTCTTGGAAGCCAATTC | | 90.6 | Tafolla-Arellano et al. 2017 ^51^ | |
| *MiCER3* | | MIN064126 | | GAGGAGCCAAGAATTGAAT | GCATGTTGCTGTAGGAGTT | | 87.2 | Tafolla-Arellano et al. 2017 ^51^ | |
| *MiGPAT6* | | MIN030144 | | AGTGCCCTTTTCGCTGAGTT | CCCACGAACTGTTGTCCCAT | | 95.6 | This study | |
| *MiCUS1* | | MIN010966 | | GACAAGGACCCTACAATGGAATTGG | GATCTGTTGTACGATAACTCTGCCG | | 91.8 | Tafolla-Arellano et al. 2017 ^51^ | |
| **Periderm related** | |  | |  |  | |  |  | |
| *MiMYB93* | | MIN065836 | | GTGGAAAGAGCTGCCGTCTA | GGACCACTTGTTCCCCAGTAT | | 93.1 | This study | |
| *MiNAC058* | | MIN000125 | | TTCACAAGGGCACAGGGATC | CCGGGTTTGCTGGAGTTGTA | | 94.5 | This study | |
| *MiCYP86A1* | | MIN017715 | | GGCCACAGTGTTCCGTTTTG | GCCGCCTGATAACTTCCTCA | | 75.8 | This study | |
| *MiCYP86B1* | | MIN017715 | | TAGGCTGTTACCCGTGCTTG | GGGGTCAACTCCGAATGCAA | | 108.2 | This study | |
| *MiGPAT5* | | MIN103548 | | TGTCACGTTTATTTGGCGGC | TGCATACAAAGAGGACCCCG | | 86.1 | This study | |
| *MiABCG20* | | MIN015881 | | CCCACTTTTGCCAATCCGTT | TCCGGTTACCAAAACTGCCC | | 93.3 | This study | |
| **Reference genes** | |  | |  |  | |  |  | |
| *MiActin1* | | JF737036 | | CGTTCTGTCCCTCTATGCCA | AGATCACGGCCAGCAAGATC | | 88.3 | Luo et al. 2013 ^52^ | |
| *MiTUBB* | | OP047693 | | TCGTCTATGATGGCTAAGTGTGA | AGTTGGTGGCTGGTAGTTGATA | | 85.7 | Yao et al. 2022 ^53^ | |
|  | |  | |  |  | |  |  | |
